# Self-Regulation in Children with Neurodevelopmental Disorders “SR-MRehab: Un Colegio Emocionante”: A Protocol Study

**DOI:** 10.3390/ijerph17124198

**Published:** 2020-06-12

**Authors:** Dulce Romero-Ayuso, Pablo Alcántara-Vázquez, Ana Almenara-García, Irene Nuñez-Camarero, José Matías Triviño-Juárez, Patrocinio Ariza-Vega, José-Pascual Molina, Pascual González

**Affiliations:** 1 Department of Physical Therapy, Occupational Therapy Division, Faculty of Health Sciences, University of Granada, Avda. De la Ilustración nº60, 18016 Granada, Spain; pablo.alcantaravazquez@gmail.com (P.A.-V.); anaissabel95@gmail.com (A.A.-G.); lloverire@correo.ugr.es (I.N.-C.); pariza@ugr.es (P.A.-V.); 2 Primary Care Center Zaidín Sur, Granada Metropolitan Sanitary District, 18007 Granada, Spain; jmtjuarez@hotmail.com; 3 Biohealth Research Institute (ibs.GRANADA), Physical Medicine and Rehabilitation Service, Virgen de las Nieves University Hospital, Jaén street, s/n, 18013 Granada, Spain; 4 LoUISE Research Group, Computing Systems Department, University of Castilla-La Mancha, 02071 Albacete, Spain; JosePascual.Molina@uclm.es (J.-P.M.); pascual.gonzalez@uclm.es (P.G.); 5 CIBERSAM, Biomedical Research Networking Centre in Mental Health, 28029 Madrid, Spain

**Keywords:** emotional regulation, executive functions, ADHD, ASD, children, virtual reality

## Abstract

Self-regulation refers to the ability to control and modulate behavior, and it can include both emotional and cognitive modulation. Children with neurodevelopmental disorders may show difficulties in self-regulation. The main objective of this study is to improve self-regulation skills in children between 6 and 11 years of age with neurodevelopmental disorders. Methodology: A randomized controlled trial will be conducted with the use of “SR-MRehab: Un colegio emocionante”, based on a non-immersive virtual reality system where virtual objects can be managed by children in a natural way using their hands. Children will be recruited from several schools of Granada (Spain) and they will be randomly allocated to two groups. An assessment will be conducted before and after the intervention and 24 weeks after the end of the intervention process. The experimental group will receive the intervention using virtual reality. The control group will receive a standard self-regulation program. Both interventions will be performed once a week for a total of 10 sessions. Changes in self-regulation, as well as the acceptability of technology with the use of SR-MRehab, will be evaluated. The results will be published and will provide evidence regarding the use of this type of intervention in children with neurodevelopmental disorders. Trial registration: Registered with code NCT04418921.

## 1. Introduction

Improving the social and emotional well-being of children in vulnerable situations is a priority objective in Mental Health programs [1]. Self-regulation can be understood as one of the most complex and important executive functions, being essential for harmonious development and adequate academic progress [2,3]. It is considered to have at least two dimensions: emotional self-regulation and cognitive self-regulation. Emotional regulation refers to the ability to control and modulate emotional expressions (positive or negative) and to interact with others in increasingly complex ways according to social rules, adapt to emotionally challenging situations, and inhibit inappropriate behaviors [4]. In this sense, emotional regulation is the ability to influence your own responses, and involves perceiving what we feel, and expressing it appropriately [5]. In the case of children with neurodevelopmental disorders, such as autism spectrum disorders (ASD) [4,6], and attention deficit hyperactivity disorders (ADHD), emotional regulation and the improvement of executive functions are therapeutic aims [7,8]. Although children with autism are known to have difficulties identifying, expressing, and regulating their emotions, and displaying negative emotions, which can be reflected in irritability, challenging or aggressive behaviors, few treatment programs have focused on improving difficulties with emotional regulation [4]. ASD has been explained from different theoretical proposals, such as the difficulties of these children to mentalize, that is, to put themselves in the other’s place, and to consider that the other is a being with imagination, thoughts, feelings and intention [9]. It has been proposed that the origin of the difficulties in children with ASD is executive dysfunction, with a lack of flexibility, perseverance, stereotypes, difficulty in initiating new actions and sequence actions. Thus, for example, a difficulty shown by children with ASD is anticipation, which is related to the understanding of communicative intention, with the establishment of schemes or scripts on actions, declarative memory, in general, and prospective memory, in particular. Children with ASD have difficulties with imitation and communication skills, and show interests that are similar to those of other children their age, through symbolic play and mental representation or ideation. They may need supervision to play, which requires direct contact with an adult. This makes it more difficult for ASD children to socialize [10,11].

On the other hand, attention deficit hyperactivity disorders (ADHD) are characterized by the presence of a persistent pattern of inattention and/or hyperactivity and impulsivity that interferes with function or development. In general, they show difficulties in relationships with their peers, especially due to their impulsiveness, i.e., lack of inhibitory control, which prevents them from making a correct reading and emotional interpretation of non-verbal messages. Furthermore, it has been indicated that children with ADHD show more negative affect, greater emotional reactivation and less empathy than children with neurotypical development, and deficits in emotional self-regulation, which increase the risk of functional decline in daily life and comorbidity in these children [3]. ADHD has mainly been explained as a disorder of neurodevelopment of the prefrontal lobe, which affects the development of executive functions in childhood, including inhibitory control, working memory, cognitive flexibility, planning, the performance of goal-oriented behaviors and behavioral self-control [12,13,14]. Recent studies show that deficits in executive functioning and emotional regulation are interconnected, especially since inhibitory control, reasoning, planning and problem solving are prerequisites for emotional regulation [14,15]. Some authors have indicated that cognitive processes underlying executive functions could be understood as “*cold*” executive functions, while those involved in emotional regulation could be considered “*hot*” executive functions [16,17]. Despite this, few studies have addressed emotional regulation and executive functioning together [14].

The use of new technologies, including virtual reality (VR) and gesture recognition, reveals itself as a promising means in rehabilitation programs, in general [18], and occupational therapy, in particular [19], not only for the attractiveness and motivation that it produces in the participants, but also for all the resources that allow therapists to use such technologies, facilitating the registration of each session and the adjustment of the personalized feedback that can be offered [20]. Furthermore, virtual reality allows simulating scenarios of daily life, in such a way that we can configure them as a laboratory to develop the necessary skills in a progressive way for the most adaptive behaviors possible [21,22,23,24]. Virtual reality systems provide a sense of presence and immersion; that is, they allow us to learn skills in a safe and controlled environment; at the same time, they are usually very motivating environments, particularly for children with ASD, if the designs take into account their sensory preferences [25].

Some studies have shown the usefulness of virtual reality systems to improve emotional and social skills in children with ASD compared to traditional therapies [24,26]. VR allows us to learn skills in a safe and controllable environment in a repetitive way, which makes it an interesting tool for children with ASD, facilitating the understanding of activities, their anticipation and their own emotional regulation before the treatment. In this sense, different studies have shown that children with ASD treated with VR improve in the identification of emotions, emotional expression and regulation [27,28] and their skills for social interaction, such as starting, participating and ending a conversation [29]. Regarding the specific field of self-regulation, most works have focused on training in social skills, through the use of avatars and social scenarios, with immersive virtual reality. Moreover, improvements in cognitive flexibility and the recognition of emotions, facial expressions and body gestures have also been observed [24]. Lastly, a recent research line has begun on the usefulness of VR to differentiate the body movements of children with ASD in imitation tasks [30]. 

However, there are fewer studies addressing the intervention in children with ADHD [31]. In these cases, they are fundamentally aimed at improving executive functioning. Specifically, studies focus on improving working memory and attention control [31,32,33]. Most of the virtual reality studies with children with ADHD have focused on the recreation of a virtual classroom for the assessment of attention [34,35]. There are recent studies that use augmented reality (AR) through the empowered brain system in ADHD and ASD, with the aim of improving social interaction [36,37]. Although it has been indicated that VR may be a promising tool in intervention in children with ADHD [23], to the best of the authors’ knowledge, there is no study aimed at improving self-regulation in children with ADHD through virtual reality systems.

Despite an increase in the number of published studies on the subject, a number of limitations have been identified in previous studies. Firstly, it has been indicated that an important limitation is the lack of studies with control groups or the mere inclusion of a control group formed by healthy children and randomized controlled trials (RCTs) [25]. Another limitation of previous studies is that a large number of them only include male children and, when they refer to autism, most of them only include children diagnosed with Asperger’s or high autistic functioning. Thirdly, it has been indicated that most studies lack the incorporation of executive tasks and their application to everyday situations, in family contexts, and as realistic as possible for the child [24]. In addition, some studies show difficulties or rejection by some children with ASD to use VR glasses and head-mounted displays (HMDs) and avoid the high cost of setting up a cave automatic virtual environment (CAVE) in educational centers [38]. Finally, there are few studies of intervention programs aimed at simultaneously improving executive functioning and emotional regulation in children with neurodevelopmental disorders [24,39]. Furthermore, these interventions are reduced to the clinical setting and there are few studies that approach the intervention in the natural context of the child, such as the educational context.

### 1.1. Aims and Hypotheses

#### 1.1.1. Primary Objective

The main aim of this study is to evaluate and compare the effect of a standard self-regulation program and “SR-MRehab: Un colegio emocionante”, involving VR on emotional regulation and cognitive regulation with neurodevelopmental disorders.

#### 1.1.2. Hypotheses

Ten sessions of “SR-MRehab: Un colegio emocionante” involving VR will have the same effects as the standard program on the improvement of self-regulation.

## 2. Materials and Methods 

### 2.1. Study Design and Participants

“SR-MRehab: un colegio emocionante” is a randomized controlled trial; a non-immersive virtual reality system that will be conducted using the MRehab tool [40,41]. Children will be recruited from several schools of Granada (Spain) and they will be randomly allocated to two groups: experimental or control group. An assessment will be conducted before and after the intervention and 24 weeks after the end of the intervention process. The experimental group will receive the intervention using virtual reality. The control group will receive the intervention through a standard self-regulation program. Both interventions will be performed once a week for a total of 10 sessions (50-min sessions per day). The inclusion criteria establish that the participants must: (1) have special educational needs; (2) show oral comprehension, with the capacity to fix the gaze; (3) be able to pay attention to verbal and visual instructions; and (4) pay sustained attention for at least four minutes. The exclusion criteria are: (1) severe motor disability; (2) children with major behavioral problems, such as self-harm; and (3) children with high abilities. Initially, all students of this center with special educational needs will be invited.

### 2.2. Procedure and Enrollment

**Figure 1:**
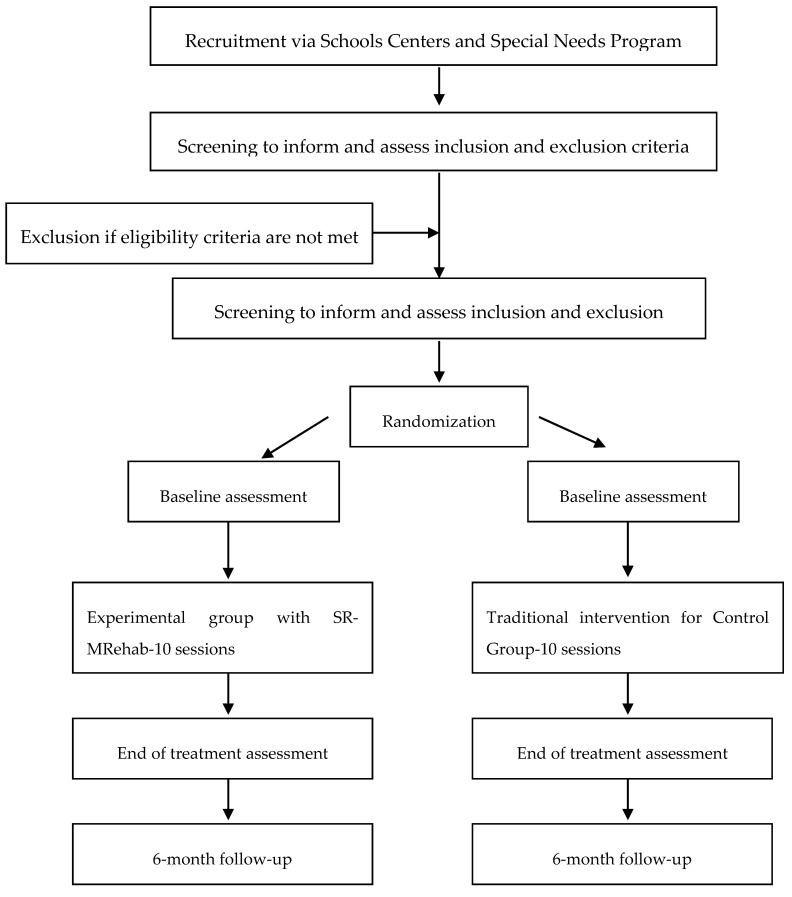
Recruitment and study procedure.

The implementation of the program covers the period from September 2020 to May 2021, divided into six stages: recruitment, pre-evaluation, allocation, intervention, post-evaluation and follow-up at 6 months. The initial stage corresponds to the acceptance by the management of the centers, and the subsequent invitation to the participants, by sending a letter to the parents/legal guardians, which contains an informative document of the characteristics of the project and the corresponding informed consent to participate in it. Children will be randomly assigned through the online randomization program. The type of allocation to each group will be simple randomization. The recruitment and study procedure are shown in Figure 1.

#### 2.2.1. Study Variables 

The main variables of the study can be divided into two main dimensions: (1) cognitive regulation or executive functions: inhibitory control, flexibility, planning, reasoning, and problem solving; (2) emotional regulation: emotional perception and emotional regulation. These variables were, and will be, evaluated through several questionnaires provided to parents, teachers and students and tests performed by children, as shown in Table 1.

Additionally, other sociodemographic data and information about the child’s development and learning history will be collected. Through the “SR-MRehab: Un colegio emocionante”, in each session, the following information will be collected for each task: number of hits, cognitive errors, and the time required by each participant to complete the task.

#### 2.2.2. Pre-Assessment, Post-Assessment and Follow-Up at 6 Months

The instruments that will be used to perform the assessment are described below.

EQ-i: YV [42] is a questionnaire designed to evaluate socio-emotional intelligence in children from 6 years of age. It contains 5 dimensions: intrapersonal intelligence, interpersonal intelligence, stress management, adaptability and general mode. It has been validated with a Spanish population and has shown acceptable psychometric properties, with a Cronbach’s alpha between 0.63 and 0.80 for the different dimensions of the questionnaire.

The NEPSY- II Battery has good psychometric properties and has been adapted and validated with a Spanish population [43]. It allows one to determine neurocognitive development in children and adolescents between 3 and 16 years of age. This tool consists of four subtests: Clock, Design Fluency, Affect Recognition, and Theory Mind. In the Clock subtest, the child is asked to draw a clock, with or without cues. Low scores on this subtest indicate deficits in planning and organization skills, poor visual space ability to draw or read, and poor time perception. The Design Fluency subtest is a non-verbal fluency task in which the child must draw as many unique designs as possible in a time limit, from an initial structure or without structure. Poor performance in this task is related to difficulties in initiating actions and poor cognitive flexibility. In the Affect Recognition subtest, the child is asked to recognize different emotions in different photographs, both freely and in association with other faces. Lastly, in the Theory Mind subtest, the child is shown different photos and asked what he/she thinks the people in the photo are experiencing.

EPYFEI and EPYFEI-Escolar are two questionnaires for parents and teachers, respectively, for the evaluation of children between 3 and 11 years of age. Both have been developed and validated with a Spanish population, with good psychometric properties. These questionnaires can discriminate between different neurodevelopmental disorders in children and allow determining which factors of executive functioning and sensory processing may be most affected. They can both be downloaded from the website enabled by the University of Granada (https://digibug.ugr.es/handle/10481/57781).

The Trail Making Test (TMT) is a neuropsychological test aimed at evaluating attention, psychomotor speed and cognitive flexibility. The Stroke test consists of two parts: part A, in which the child is asked to link 25 numbers in ascending order, arranged disorderly on a sheet. In part B, the child is asked to list the numbers and letters on the page in ascending order and according to the alphabet, that is, number-letter-number-letter. It also has good psychometric properties and different formats adjusted to the age of the child [44].

The Stroop test is widely used in the neuropsychological examination of attention, especially to determine the difficulties in interference inhibition. It has been validated with a Spanish population and is usually employed from the age of 6, or when children have developed good reading skills. For younger children, other formats are available. This test also has good psychometric properties [44].

The assessment will be conducted in the first weeks of December 2020, divided into three blocks. Firstly, the EQ-i:YV questionnaire [42] and the Clock and Affect Recognition subtests of NEPSY-II [43] will be administered to the participants; separately, the teachers and parents will be asked to complete the EPYFEI-Escolar [45] and EPYFEI [46] questionnaires, respectively, which they will have to fill in and give back within the next week. Then, the participants will complete the Design Fluency and Theory of Mind subtests of NEPSY-II. Finally, in a third session, they will carry out a final block to assess executive functions through Trail Making Test A and B (TMT A-B) and Stroop test. 

The same evaluation will be carried out immediately after finishing the program. In addition, the acceptability of the technology will be evaluated with the children at the end of each session and at the end of the whole program. A combination of methods will be used to assess acceptability [47]. Firstly, in each session, each activity will be evaluated using a Smileyometer, which is a visual analog scale with a coding based on a 5-point Likert scale (Horrible = 1; Great = 5). In addition, at the end of the program, the method “*This or That*” will be used, using five questions, for example: ‘What part of the program did you enjoy the most?’, ‘What game would you like to have at home?’ To check the long-term effects of the program, a follow-up will be carried out at 6 months, in which the emotional regulation and executive functioning of the participants will be re-assessed.

#### 2.2.3. Intervention

##### Experimental Group 

The intervention on self-regulation will be carried out through a non-immersive virtual reality platform, “SR-MRehab: Un colegio emocionante”, in which the participants must conduct a series of activities designed specifically for this purpose. These activities will be performed by the children using mainly their hands to manage the virtual objects shown in the screen. To this end, our system makes use of a Kinect motion sensor connected to the computer, to control the body movements of the child. Moreover, our system records some relevant data of the execution of these activities for further analysis of the child’s performance.

The tasks will be divided into two blocks: emotional regulation (ER) and cognitive regulation (CR) (or executive functions), in a total of 10 sessions, once a week, performing a task of each block per session. Each session will last 50 min and will generally take into account the development of the different executive functions (working memory, inhibitory control, flexibility, reasoning, planning and problem solving) [15] and emotional competencies (emotional perception, and emotional self-regulation) [48]. Table 2 and Table 3 show the organization, description and aims of each session for the Experimental and Control Group, respectively. These sessions will take place from January to April 2021.

The principles of the program will be based on the following premises for children with ASD [7,49]: (1) strengthening communicative attempts; (2) choosing the most natural contexts possible; (3) establishing structured and predictable routines and environments; (4) visually supporting information; and (5) adapting our language to the understanding level of the child. Regarding the difficulty of interpreting and responding to social emotions and cues, perspective games are of great interest, where the child and the therapist describe the same object from different perspectives, as well as guessing games (guess what is in this box) and games with social stories, which are short stories that use pictograms to help understand situations with social ambiguity: (1) describing what happens; (2) orienting the action, indicating the child what to do in that situation; (3) anticipating and explaining what can happen and how the child can feel; and (4) developing a strategy that can be applied in similar situations. Virtual stories narrated with puppets can be used in young children. In older children, training techniques may also be used in social skills, with role-playing, theatre, or body expression groups. These activities can be very helpful in teaching skills to predict people’s most likely behavior in certain situations, explaining how people feel, recognizing emotions of joy, sadness, fear, anger, etc., predicting those emotions based on the situation, understanding that emotions depend on desires and beliefs, and assuming someone else’s perspective, which may not match one’s own perspective.

**Figure 2:**
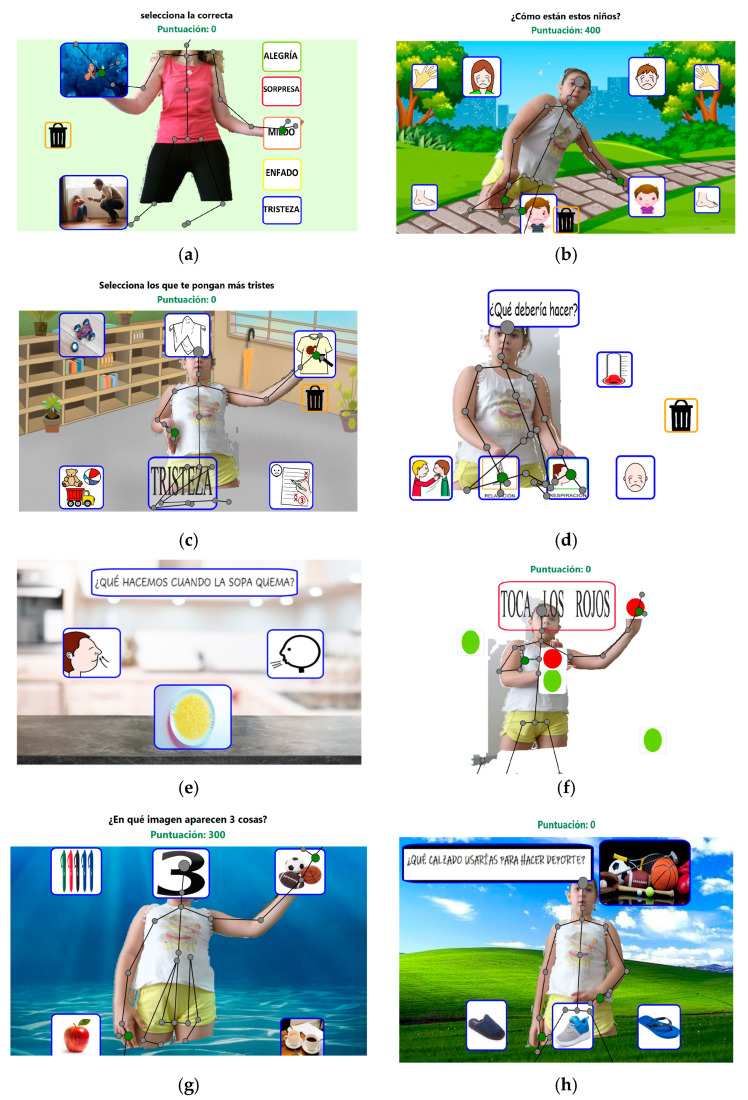
Example of tasks with “SR-MRehab: Un colegio emocionante”: (**a**–**c**) Emotional Perception, (**d**,**e**) Emotional Regulation; (**f**) Cognitive Regulation: inhibitory control; and (**g**,**h**) Cognitive Regulation: Reasoning and problem solving.

Regarding the executive functions, the proposed activities are intended for the children to notice and detect small changes in the virtual setting, learn a search strategy or visual scan, formulate an action plan, inhibit automatic responses, inhibit distractors to the task (such as noises or irrelevant stimuli for the execution of the activity), increase the maintenance of attention, monitor the activity and the steps taken, predict the consequences of actions, consider alternatives, etc. An example of the tasks is shown in Figure 2.

The MRehab tool allows one to establish five levels of difficulty. Before starting the intervention program, a database will be created with each participant, assigning each of the tasks designed for each child according to the results of the previous assessment.

The intervention will begin with the explanation of the group session of 3 to 4 children. Subsequently, to record the responses and perform the activities, the participants will be asked to perform different tasks, with the support and supervision of the occupational therapist and psychologist. The session will end with a small assembly in which each child discusses his/her experience and how and where the skills learned could be generalized in their activities of daily living. Each session will last one hour.

##### Control Group

The children from the control group will follow a program of emotional education for primary schools, through group activities in the classroom [5,50]. Each session will last 50 min, as in the experimental group. The content of the sessions will include 5 sessions of emotional awareness and 5 sessions of emotional and cognitive regulation. The activities will be similar to those listed in Table 2 for the experimental group, although the virtual reality system will not be used (see Table 3). These sessions will be conducted in parallel in a different room of the school, on the same day and time, carried out by occupational therapists and students from the last year of the degree in occupational therapy.

#### 2.2.4. Outcome Measures

##### Primary Outcome Measure: Emotional Regulation

(1) Emotional Perception

According to the previously presented aims and hypotheses, after performing the intervention protocol, the participants in the experimental group are expected to change in the number of errors in the recognition of recognized emotions on faces in the Affect Recognition subtest of the Children’s Neuropsychology Assessment Battery (NEPSY-II), in which the minimum scores are “0” and the maximum number of errors for children older than 6 years is 79. The higher the score, the worse the recognition of emotions.

(2) Emotional Regulation

The participants in the experimental group are expected to change in their ability to understand mental functions, such as belief, intention, and deficit to understand the relationship between emotions and the social context, according to the Theory of Mind subtest of the Children’s Neuropsychology Assessment Battery (NEPSY-II), in which the minimum score is “0” and the maximum score is 28. The higher the score, the better the ability to mentalize and recognize the mental states of other people.

##### Secondary Outcome Measure: Cognitive Regulation

(1) Cognitive Flexibility

After performing the intervention protocol, the participants in the experimental group are expected to change their shills of maintaining attention and for the ability to change and maintain a new pattern of responses, according to the Auditory Attention and Cognitive Flexibility Subtest of the Children’s Neuropsychology Assessment Battery (NEPSY-II), in which the minimum score is “0” and the maximum score is 30. The higher the score, the number of correct answers, the better the ability to pay attention and to show a flexible behavior with changes. In addition, these subtests allow us to know the number of errors, divided in three types: commission, omission and inhibition errors. The greater the number of errors, the worse the attention and cognitive flexibility. The minimum error score is “0” and the maximum number of commission errors is 189; the maximum number of omission errors is 39 and the maximum number of inhibition errors is 33.

(2) Planning and Sequencing

The participants in the experimental group are expected to change in planning and organization skills, as well as in self-supervision skills, according to the Clocks subtest from Children’s Neuropsychology Assessment Battery (NEPSY-II), in which the minimum score is “0” and the maximum score is 78. A higher score means better skills to plan, sequence and monitor the action.

### 2.3. Data Mangement

The data will be collected from the results obtained in the assessment tests and questionnaires that will be provided to the parents and teachers, being subsequently introduced in SPSS, assigning each student a confidential code based on their school year and always respecting their anonymity. The data manager will create the database in SPSS, debug the database and perform all data analyses. The data manager will be blinded to the randomization of the participants.

### 2.4. Statistical Analysis

#### 2.4.1. Sample Size

According to the results of the study by Negut, Jurma and David [35], who evaluated the attention in children with ADHD using an AULA VR (virtual reality classroom) system, the minimum standardized difference (d/s, where d = difference between neurotypical and clinical groups; s = standard deviation of the neurotypical group) that could be considered clinically relevant is one point. According to this value, for a bilateral contrast with α = 0.05, and a power of 1-β = 0.90, the initially estimated sample size is 21 children. Taking into account the possible losses during the evaluation, the sample size will be increased by 20%, thus, the estimated final sample size is 26 children with neurodevelopmental disorders.

#### 2.4.2. Data Analysis Plan

A descriptive analysis will be carried out for all the variables in the study. The qualitative variables will be described with absolute frequencies and percentages for each of their categories, and quantitative variables with mean, standard deviation (DE), maximum and minimum values, as well as median and interquartile range, where appropriate. The corresponding 95% confidence intervals (ICs) will be calculated. The normality of the continuous variables will be verified using graphical methods and normality tests.

The Chi-square test will be used to study the statistical significance of the differences between proportions. To study the statistical significance of the differences between means, different tests, questionnaire scores, completion time and number of errors of each virtual task throughout the different sessions, the following tests will be used depending on whether or not the variables to be compared show normality: Student’s t test, Mann–Whitney U-test, ANOVA or Kruskal–Wallis test. Furthermore, the effect size of the changes observed will also be calculated. The analysis of the association between two quantitative variables will be performed, using Pearson’s or Spearman’s correlation coefficient, depending on whether or not the variables show normality.

If necessary, generalized linear regression models will be used to adjust the results for any confounding covariate that shows significant baseline differences between the groups and a correlation with the result. For the hypothesis testing, a significance level of 5% will be assumed (*p* < 0.05). All statistical analyses will be carried out using the IBM SPSS software (version 24, IBM Corporation, New York, NY, USA).

### 2.5. Ethical Approval, Ethical Considerations and Dissemination

The study protocol was approved by the Research Ethics Committee of the University of Granada (registration number 1018/CEIH/2019), and this study was registered at ClinicalTrial.gov (under identification no. NCT04418921). This study respects the principles established in international and national legislation in the field of biomedicine, biotechnology and bioethics, as well as all the rights derived from the protection of personal data. All investigators will have updated their report of criminal offences, as required by the current legislation for working with children in Spain.

The entire educational community will be informed about the project verbally and in writing. Participation in the project will be voluntary and all participants will give their written informed consent and will not receive any incentive to participate in the study. Weekly, the parents, teachers and the educational community, in general, will be informed about the progress of the project, through the school blog. The results of this study will be presented at international scientific meetings and will be disseminated in scientific journals. In addition, this study will be the subject of a Master’s thesis (TFM) and a final degree project (TFG).

## 3. Discussion

The use of VR as a therapeutic means is increasing in childhood, especially for the treatment of pain in children with cancer [51] and anxiety before dental treatment [52,53]. In the case of neurorehabilitation, different studies address it in cerebral palsy and Down’s syndrome to improve motor control, gait, and balance [54,55], and in the field of childhood mental health to treat anxiety (social phobia, school phobia) and eating disorders [56].

This study will investigate training to improve emotional regulation and executive functioning in children with special educational needs (ADHD and/or ASD) through the “SR-MRehab: Un colegio emocionante” program with non-immersive virtual reality. In recent years, increasing interest has been observed in the use of VR in children with neurocognitive disabilities, especially in the area of autism. However, some studies with VR have found that children with ASD may have more difficulties accepting the use of head-mounted displays (HMDs) or other devices. For this reason, non-immersive virtual reality or AR systems may be a better option in these cases [29]. In fact, it has been indicated that non-immersive VR or AR could improve the quality of sensory experiences [25], with this being a more critical aspect in some disorders such as ASDs than in those presenting sensory hypersensitivity [57,58,59]. The technology that is proposed to be used in this protocol is a tool based on a low-cost non-immersive virtual reality system. In this sense, a study recently tried to find out what type of virtual reality systems in classrooms seemed more attractive to children with ASD. Their results showed that these children were interested in using virtual reality again, to feel more relaxed or calm [38].

In our study, the results of a traditional educational program for the development of self-regulation (control group) and a program designed using our non-immersive virtual reality system (experimental group) will be compared. The effectiveness of the treatment will be evaluated through direct tests with the children and questionnaires about their participation and performance in their activities of daily living and in the classroom. The children will be assessed before and immediately after the intervention. Many interventions do not address emotional regulation and cognitive regulation together. The proposed protocol will evaluate the effectiveness of the proposed virtual reality tool “SR-MRehab: Un colegio emocionante”, versus standard program without VR.

At the time of submission of this manuscript (5 May 2020), the project has been presented to the educational community of “Parque de las Infantas” Infant and Primary School. The project is expected to start in October 2020. One possible limitation of this study is the type of sampling, which is convenience, voluntary and non-probabilistic.

Another potential limitation is related to the fact that the improvement found in emotional regulation and executive functioning might not only result from the program itself, but also from the natural development that occurs in the months in which the intervention is carried out. However, these changes should be reduced, due to the scattering of the 10 intervention sessions established over the study period of six months.

## 4. Conclusions

The present study will provide data on the effectiveness and acceptability of the “SR-MRehab: Un colegio emocionante” intervention, as well as data from feasibility trials to provide information about the design and sample size for a large-scale trial. In addition, based on the experiences of the participants in the intervention, qualitative data will be obtained to determine the feasibility of the MRehab tool [25,41], to design and perform these rehabilitation activities in a school setting.

## Figures and Tables

**Table 1:** Variables and outcome measures.

Variables	Dimension/Processes	Instruments		Participants		Baseline	During Intervention	Immediately Post-Intervention	24 Weeks Post-Intervention
			Parents	Teachers	Students				
Gender Age Special Needs Diagnosis			X X X	X		X X X X			
Parent’s Educational level			X						
Previous experience with VR and serious games			X		X				
Emotional Regulation	Emotional Awareness	NEPSY-II: Affect Recognition			X	X		X	X
	Emotional Awareness	NEPSY-II: Theory of Mind			X	X		X	X
	Self-regulation	EPYFEI	X			X		X	X
	Self-regulation	EPYFEI-Escolar		X		X		X	X
	Emotional Competencies	EQ-i: YV *			X	X		X	X
Cognitive Regulation/Executive Functions	WM, IC. Pln, Sq	EPYFEI	X			X		X	X
	WK, IC, Pln, Sq	EPYFEI-Escolar		X		X		X	X
	Pl. Sq, Fl	NEPSY-II: Design Fluency			X	X		X	X
	Pl, Sq	NEPSY-II: Clock *			X	X		X	X
	Executive Attention	Trail Making Test A & Trail Making Test B			X	X		X	X
	IC	Stroop Test			X	X		X	X
	Virtual Tasks SR-MRehab	Percent Completed Nº of Errors Type of errors Activity Time Time without interaction Reason for non-attendance or early withdrawal					X X X X X X X		
		Acceptability			X			X	

* Only for children between 7 to 12 years of age. WM: working memory, IC; inhibitory control; Pln: Planning; Sq: Sequencing; Fl: Flexibility.

**Table 2:** Organization, description and aims of each session for Experimental Group.

Variables/Aims Session	Emotional Regulation	Aim	Description	Cognitive Regulation/Executive Functions	Aim	Description
1	Do you know the emotions?	Emotional perception	In the virtual school environment, children must identify the emotions on the faces of different children and adults.	Figure selection	Inhibitory control Working memory Flexibility	This activity consists in the selection of geometric figures that will appear on the left/right or top/bottom of the screen. The figures are organized in pairs, and the objective is to select the correct item from the appearance or disappearance of a signal. In the first two levels, an arrow indicates which option is correct. In the third and fourth levels, pairs of arrows will initially appear, one of which will then disappear, with the remaining one indicating the correct answer. In the fifth level, the signal will be a colored star (red or green), which will indicate the option to select. Finally, at level 6, the mechanism is similar to that of the third and fourth levels, that is, pairs of stars of different colors (red and green) will appear, of which one will disappear, with the color of the remaining one indicating the correct answer of the two options
2	Match and differences	Emotional perception Theory of Mind	Different family scenarios are presented at school and children are asked to identify the emotions on the faces and those shown by the body, in order to stimulate mentalization. What is this child thinking? How does the child feel? Do you choose a similar emotion in these two scenarios? Do you select contexts and situations where you can feel the same way?	Select equal and opposite emotions	Inhibitory control Working memory Flexibility	This activity is similar to the previous one, with the exception that this one is focused on emotions.
3	How are you? Virtual Emotion-Meter	Emotional perception	First, the child must identify or select how he/she feels and then indicate the intensity of the emotion.	Red No, green Yes: Traffic light One	Inhibitory control Working memory Flexibility Reasoning	Simulating a traffic light, it is explained that red indicates that we must stop before acting, for example, when we do not know the solution of a task, or at a certain indication, for example, from the teacher, such as when we cross a street. The yellow color means that we must be careful, think before acting (How can I solve the problem or situation?), and green can be applied when we know the solution to a problem very well. Different everyday situations arise, which pose a challenge for the child to inhibit impulsive behavior. The child must identify each red, yellow or green situation based on his/her own characteristics and situations in real life. Also, when a red signal comes out, the child cannot move. Otherwise, it will be counted as an error of commission.
4	How is your engine?	Emotional perception	In this task, the child has to indicate how calm or nervous he/she feels, and the intensity of such feeling. Finally, he/she is asked to think about how he/she can feel calmer and select among the different options to relax and feel good.	Simon says	Inhibitory control Flexibility	In this task, the child is asked to follow the instruction on the screen, only when the command is “Simon Says”. Upon any other request, that is, without the “Simon Says” command, the child must not move, in order to work the inhibitory control.
5	The Volcano	Emotional perception and regulation	The child has to indicate how angry or happy he/she feels. Finally, he/she is asked to think about how he/she can feel happier, how to generate positive feelings and select among the different alternatives to relax and feel good.	Find the difference!	Inhibitory control Flexibility Reasoning Problem solving	This task is divided into two blocks of 5 levels each. In the first block, a number from 1 to 5 will appear, along with several images containing different amounts of objects (1 to 5). The task is to match the indicated number with the image that represents that number. In the second block, all the numbers will appear, and, again, images with different amounts of objects. The task is to re-match each number that appears on the screen with the quantity that it represents.
6	Emotional traffic light	Emotional regulation	Simulating a traffic light, it is explained that red indicates that we must stop before acting, for example, when we are angry, yellow means that we have to think how we feel and how we can solve the problem or situation, and green can be applied when we are relaxed and can act calmly. The child must identify each red, yellow or green situation based on his/her own characteristics and situations in real life	Find your treasure!	Inhibitory control Flexibility Reasoning Solving problems	The child is asked to create a collage with words, short sentences and images that reflects how he/she can help other people and positive qualities of oneself. Later, the child must select actions that help him/her at home, at school, that is, what and how makes him/her feel useful and self-competent, and how others see him/her when he/she is useful.
7	Hot soup! The Lazarillo	Emotional regulation	The child must learn to relax through breathing, which is why the metaphor of hot soup is used, in which one has to blow and practice breathing. The child has to imitate the images and follow the prompts to blow the hot soup. In addition, at a later stage the child will be a “guide”, guiding the process of cooling the soup and must select the appropriate image	Looking at all sides	Flexibility Working memory	The aim of this task is for the participant to remember which animals previously appeared. To do this, an image will first be shown in which various animals will appear for 30 s, during which the patient will have to retain the displayed information. Once prepared, the participant will press GO! To make way for a new screen where animal drawings will appear. The patient’s task will be to select those animals that appeared in the previous image and discard those that did not. The difficulty increases proportionally with the number of animals and distractors that will appear
8	Bubbles or stones?	Emotional regulation	Through a story, they must explain which stones are the negative feelings (anger, sadness...) and the bubbles are positive feelings (happy, peace, calm). The child is asked to indicate what the backpack of different children would be filled with in different situations. Later, he/she is asked to choose what he/she wants to fill his/her backpack with, that is, bubbles or stones	Discover the stones and touch the bubbles!! How’s your backpack?	Reasoning Planning Problem solving Flexibility	In this task, starting from the story of the stones and bubbles, questions will be asked and various response options will be provided, which the children must correctly select using logic. Difficulty increases through with number of correct options and their similarity to distractors
9	Glasses to think well!!	Emotional regulation	Thoughts and feelings are related. Sometimes a positive thought can make us feel good and negative thoughts can make us feel bad. The child must choose those positive thoughts that make him/her feel good and learn to use it in his/her daily life	The turtle	Working memory Inhibitory control Flexibility Reasoning Problem solving	From the story of the turtle, as a self-control strategy, in this task, the image of a turtle will be exposed to the participant, who must observe it for a given period of time, until it disappears. When the participant is ready, he/she will press GO! To be launched to a screen, where an incomplete puzzle from the previous image will appear. Different pieces appear on the right, of which only one completes the puzzle. The participant’s task is to select that piece. The difficulty increases as the participant advances through the levels, adding more pieces as options and using more complex images
10	Letter of thanks	Emotional regulation	Thanksgiving can be a way to generate positive feelings in others and in oneself. The child must select situations in which it is appropriate to say “thanks” and images that show gratitude and allow us to “write” a letter of thanks to a friend, teacher, or family member.	10 point Check-In	Inhibitory control Working memory Flexibility Reasoning Planning Problem solving	This task consists of 10 stages in a countdown sequence: (10) the children have to select 10 images that make them feel relaxed; (9) select 9 things that being with a certain letter or which can be found in a certain space (classroom, schoolyard, etc); (8) select 8 known people; (7) select 7 colors; (6) choose 6 things that make them feel happy; (5) choose images that reflect relaxation and imitate them; (4) select 4 objects they hear; (3) select 3 things that are in the room that they like to play with; (2) choose two images of techniques to relax and imitate them; (1) select the current emotion and thoughts

**Table 3:** Organization, description and aims of each session for Control Group.

Variables/Aims Session	Emotional Regulation	Aim	Description	Cognitive Regulation/Executive Functions	Aim	Description
1	Do you know the emotions?	Emotional perception	Children must identify the emotions on cards of different children and adults.	Choose a number!	Inhibitory control Working memory Flexibility	In this activity, the children have to choose a number from 1–10. Depending on the number, a card corresponds to it. Each card has a red/green right/left or up/down arrow. So the child has to take a step as indicated by the arrow when the color of the arrow is green or make the opposite movement when the color of the arrow on the chosen card is red.
2	Match and differences	Emotional perception Theory of Mind	Different pictures are presented and children are asked to identify the emotions on the faces and those shown by the body, in order to stimulate mentalization. What is this child thinking? How does the child feel? Do you choose a similar emotion in these two scenarios? Do you select contexts and situations where you can feel the same way?	Select equal and opposite emotions	Inhibitory control Working memory Flexibility	This activity is similar to the previous one, with the exception that this one is focused on emotions.
3	How are you? Emotion-Meter	Emotional perception	First, it is explained to the child that our emotions can be graded in intensity like a thermometer. Then, the child must identify or select how he/she feels and finally they must indicate the intensity of the emotion.	Red No, green Yes: Traffic light One	Inhibitory control Working memory Flexibility Reasoning	With different colored ballons, simulating a traffic light, it is explained that red indicates that we must stop before acting, for example, when we do not know the solution of a task, or at a certain indication, for example, from the teacher, such as when we cross a street. The yellow color means that we must be careful, think before acting (How can I solve the problem or situation?), and green can be applied when we know the solution to a problem very well. Different everyday situations arise, which pose a challenge for the child to inhibit impulsive behavior. The child must identify each red, yellow or green situation based on his/her own characteristics and situations in real life. Also, when a red signal comes out, the child cannot move. Otherwise, it will be counted as an error of commission.
4	How is your engine?	Emotional perception	In this task, the child has to indicate how calm or nervous he/she feels, and the intensity of such feeling. Finally, he/she is asked to think about how he/she can feel calmer and select among the different options to relax and feel good.	Simon says	Inhibitory control Flexibility	In this task, the child is asked to follow the oral instruction given by therapist, only when the command is “Simon Says he/she must do it. Upon any other request, that is, without the “Simon Says” command, the child must not move, in order to work the inhibitory control.
5	The Volcano	Emotional perception and regulation	The child has to indicate how angry or happy he/she feels. Finally, he/she is asked to think about how he/she can feel happier, how to generate positive feelings and select among the different alternatives to relax and feel good.	Find the difference!	Inhibitory control Flexibility Reasoning Problem solving	In this task, a child is given two daily scenes, the objective is to find subtle differences. To do this, the rest of the group should give verbal clues or directions if the child asks for help to solve it. Each time it is up to a child to find the differences.
6	Emotional traffic light	Emotional regulation	Simulating a traffic light, with colored cards, it is explained that red indicates that we must stop before acting, for example, when we are angry, yellow means that we have to think how we feel and how we can solve the problem or situation, and green can be applied when we are relaxed and can act calmly. The child must identify each red, yellow or green situation based on his/her own characteristics and situations in real life	Find your treasure!	Inhibitory control Flexibility Reasoning Solving problems	The child is asked to create a collage with words, short sentences and images that reflects how he/she can help other people and positive qualities of oneself. Later, the child must select actions that help him/her at home, at school, that is, what and how makes him/her feel useful and self-competent, and how others see him/her when he/she is useful.
7	Hot soup! The Lazarillo	Emotional regulation	The child must learn to relax through breathing, which is why the metaphor of hot soup is used, in which you have to blow and practice breathing. The child has to imitate the therapist and follow the prompts to blow the hot soup. In addition, at a later stage, the child will be a “guide” who will guide the process of cooling the soup to her classmates.	The right sequence!	Flexibility Working memory	The objective of this task is for the participant to pay attention to the sequences of activities, objects or animals (because they are usually significant for children). First, an image will be shown in which several objects/animals or activity sequences will appear for 30 s, during which the patient will have to retain the displayed information. When the therapist hides the images, the child should try to construct the same sequence with other similar images. The difficulty increases proportionally with the number of distractors displayed on the cards.
8	Bubbles or stones?	Emotional regulation	Through a story, they must explain which stones are the negative feelings (anger, sadness...) and the bubbles are positive feelings (happy, peace, calm). The child is asked to indicate what the backpack of different children would be filled with in different situations. Later, he/she is asked to choose what he/she wants to fill his/her backpack with, that is, bubbles or stones	Discover the stones and touch the bubbles!! How’s your backpack?	Reasoning Planning Problem solving Flexibility	In this task, starting from the story of the stones and bubbles, questions will be asked and various response options will be provided, which the children must correctly select using logic. Difficulty increases through with number of correct options and their similarity to distractors To make it more playful, it is done in a similar way to “Goose Game”.
9	Glasses to think well!!	Emotional regulation	Thoughts and feelings are related. Sometimes a positive thought can make us feel good and negative thoughts can make us feel bad. The child must choose those positive thoughts that make him/her feel good and learn to use it in his/her daily life	The turtle	Working memory Inhibitory control Flexibility Reasoning Problem solving	In this activity, children are told the story of the turtle. They are taught how a turtle does to reflect and think when she has a problem. Finally, to reinforce what has been learned, a bookmark is made with the children with the image of a turtle.
10	Letter of thanks	Emotional regulation	Thanksgiving can be a way to generate positive feelings in others and in oneself. The child must select situations in which it is appropriate to say “thanks” and images that show gratitude and allow us to “write” a letter of thanks to a friend, teacher, or family member.	10 point Check-In	Inhibitory control Working memory Flexibility Reasoning Planning Problem solving	This task consists of 10 stages in a countdown sequence: (10) the children have to select 10 images that make them feel relaxed; (9) select 9 things that being with a certain letter or which can be found in a certain space (classroom, schoolyard, etc); (8) select 8 known people; (7) select 7 colors; (6) choose 6 things that make them feel happy; (5) choose images that reflect relaxation and imitate them; (4) select 4 objects they hear; (3) select 3 things that are in the room that they like to play with; (2) choose two images of techniques to relax and imitate them; (1) select the current emotion and thoughts
